# The evolving interaction of low-frequency earthquakes during transient slip

**DOI:** 10.1126/sciadv.1501616

**Published:** 2016-04-22

**Authors:** William B. Frank, Nikolaï M. Shapiro, Allen L. Husker, Vladimir Kostoglodov, Alexander A. Gusev, Michel Campillo

**Affiliations:** 1 Équipe de Sismologie, Institut de Physique du Globe de Paris, Paris Sorbonne Cité, CNRS, 75238 Paris, France.; 2 Department of Earth, Atmospheric and Planetary Sciences, Massachusetts Institute of Technology, Cambridge, MA 02139, USA.; 3 Institute of Volcanology and Seismology FEB RAS, Petropavlovsk-Kamchatsky 683006, Russia.; 4 Instituto de Geofísica, Universidad Nacional Autónoma de México, 04150 Ciudad de México, Mexico.; 5 Institut des Sciences de la Terre (ISTerre), Université Grenoble Alpes, CNRS, IRD, 38610 Gières, France.

**Keywords:** seismology, low-frequency earthquakes (LFEs), slow earthquakes

## Abstract

Observed along the roots of seismogenic faults where the locked interface transitions to a stably sliding one, low-frequency earthquakes (LFEs) primarily occur as event bursts during slow slip. Using an event catalog from Guerrero, Mexico, we employ a statistical analysis to consider the sequence of LFEs at a single asperity as a point process, and deduce the level of time clustering from the shape of its autocorrelation function. We show that while the plate interface remains locked, LFEs behave as a simple Poisson process, whereas they become strongly clustered in time during even the smallest slow slip, consistent with interaction between different LFE sources. Our results demonstrate that bursts of LFEs can result from the collective behavior of asperities whose interaction depends on the state of the fault interface.

## Teaser

The clustering of repeating low-frequency earthquakes through interaction reveals the state of the tectonic plate boundary.

## INTRODUCTION

Bursts of low-frequency earthquakes (LFEs), defined as many repeating events whose sources are closely spaced in time and typically identified within tectonic tremor (*1*–*3*), are most often observed during slow-slip events (*4*, *5*), which are aseismic slip events along plate interfaces that are capable of releasing as much strain as megathrust earthquakes (*6*, *7*). LFEs are thought to occur on small seismogenic asperities that are embedded in a mostly creeping part of the fault interface. A simple interpretation is that these asperities are loaded and generate seismic events during episodes of slow slip. The rate of tectonic tremor or LFEs is therefore often considered as a proxy for slip rate (*4*, *8*, *9*).

### LFEs in Guerrero, Mexico

**Fig. 1:**
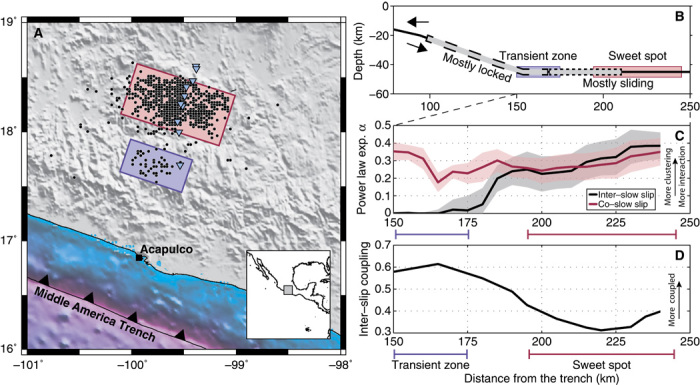
Evolution of LFE clustering in the Guerrero subduction zone. (**A**) Map view of the Guerrero subduction with all 1120 LFE epicenters shown as black points. The colored boxes represent the two LFE source regions: the transient zone in purple and the sweet spot in red (*5*, *8*). (**B**) Vertical profile of the subduction beneath Guerrero, Mexico. The two labeled colored boxes represent the LFE source regions in (A). The plate boundary transitions from a mostly locked interface to a mostly sliding one between the two regions of slow slip, represented by the two gray patches (*7*, *18*). (**C**) LFE clustering as a function of distance from the trench, as evidenced by the measured power law exponent α (see text). (**D**) Inter–slow-slip coupling along the Guerrero subduction zone (*17*).

Here, we study the relationship between slow-slip events and LFE occurrence using a catalog of 1120 LFE sources, containing more than 1.8 million individual events detected over 2 years, in Guerrero, Mexico, where the Cocos Plate subducts underneath the North American Plate (*5*, *10*, *11*). Similar to other regions where they have been observed, LFEs occur in Guerrero as small repeating shear events located along the plate interface (*2*, *12*–*15*). Previous studies in Guerrero (*5*, *8*, *16*) have established that there are two distinct source regions, as shown in Fig. 1. The “transient zone” is located at the kink where the Cocos Plate bends back toward the surface before staying subhorizontal for 250 km. This part of the interface remains locked most of the time and is activated during slow-slip events (*17*, *18*). The “sweet spot” that is located 40 km downdip from the transient zone emits bursts of tectonic tremor and LFEs nearly continuously (*5*, *6*, *16*), and is thought to be in a mostly sliding or creeping regime.

**Fig. 2:**
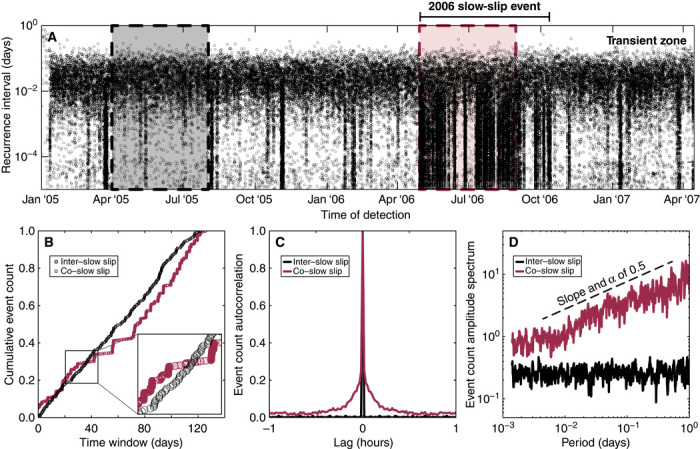
Time clustering of LFEs. (**A**) Recurrence intervals of the transient zone LFEs (see Fig. 1), defined as the elapsed time between sequential events. The two dashed boxes indicate the two time periods analyzed. (**B**) Cumulative event count of a single transient zone LFE source, whose stacked waveforms are shown in fig. S1. The zoomed inset highlights the distinctly different timing during the two time periods. (**C**) Autocorrelations of the event count signals (see text) for the events shown in (B). (**D**) Event count spectra of the two autocorrelograms in (C). The positive linear slope in log space indicates a power law distribution of event timing and corresponds to the power law exponent α.

Most of the 1120 repeating LFE sources (955) are located within the sweet spot with only 61 in the transient zone; the other 64 sources lie between the two principal source regions. The recurrence intervals for all 61 of these sources shown in Fig. 2A illustrate the clear correlation between the observed LFE activity and the slow-slip events. Most of the transient zone LFEs are grouped into bursts (seen as vertical strips in Fig. 2A), with most of them occurring during the strong moment magnitude (*M*_w_) 7.5 slow-slip event between May and October 2006 (*7*). The rest of the LFE bursts correspond to smaller slow-slip events that occur approximately every 3 months (*18*). In addition to the bursts, we observe a considerable background LFE activity when the transient zone remains locked. We verify that these inter–slow-slip LFEs are not false detections by comparing the stacked waveforms of the inter–slow-slip events and the co–slow-slip events for each LFE source, and find that they are extremely similar (*11*). In the following, we compare the LFE occurrence statistics during two 4-month windows shown in Fig. 2: (i) an inter–slow-slip period between April and August 2005 when no slow slip occurs and (ii) a co–slow-slip period between May and September 2006 during the large *M*_w_ 7.5 slow-slip event.

## RESULTS

### Quantifying the clustering of LFEs

The LFE occurrence at an individual asperity can be considered as a discrete time process characterized by its set (or catalog) of event times. By looking at cumulative numbers of events during two selected 4-month windows for one LFE source (Fig. 2B), one can clearly see distinctly different behaviors. During the inter–slow-slip period, the events occur at a more or less constant rate, whereas during slow slip, they are grouped into bursts, which are seen as fast jumps in the cumulative number of events. A close look at these jumps shows that they have different sizes, with large bursts often being composed of many smaller ones. This behavior hints at a scale-independent time clustering. We refer to clustering in this study in its most general sense: the timing of any given event is dependent on events that come before and after it and is not randomly distributed in time. A burst of events, therefore, does not necessarily have to be clustered, and could otherwise reflect an accelerated event rate whose timing is drawn from a random or Poisson distribution. We apply a point process formalism (*19*) to quantitatively characterize this clustering.

First, we translate the catalog for each source into a discrete event count signal with regular time steps of 1 min, binning each cataloged event into the time step during which it is observed. We then compute an autocorrelation function for this time series. Figure 2C shows the autocorrelations computed from the catalogs presented in Fig. 2B. For the inter–slow-slip period, this function looks like a Dirac at zero lag showing that the LFEs occur as a Poisson process. On the other hand, a smooth falloff from zero lag observed during slow slip indicates clustering and short-term correlations between events. Finally, we compute the Fourier spectrum of the event count autocorrelation. During the inter–slow-slip period, the amplitude spectrum is flat (Fig. 2D), as expected for a Poisson process. During slow slip, the spectral amplitude linearly increases with period (in log-log space), indicating scale-invariant time clustering. The degree of clustering is quantitatively characterized by measuring the power law exponent α, which is equal to the linear spectral slope (in log-log space). Values of α close to zero indicate a Poisson-like occurrence of events that are uncorrelated in time, whereas an α significantly larger than zero corresponds to a strongly clustered time process.

### Evolution of clustering during the slow-slip cycle

We measure the power law exponent α for each of the 1120 LFE sources during the two selected 4-month windows. To obtain a measure of α as a function of distance from the trench, we compute a 10-km moving average along the plate interface over the individual LFE sources for the two time periods (Fig. 1C). During the inter–slow-slip period, we observe a smooth transition of α from a random Poisson occurrence of events in the transient zone to a strongly clustered distribution in the sweet spot. We note that the level of the LFE clustering during this period is anticorrelated with the degree of coupling deduced from geodetic data (Fig. 1D) (*17*). During the 2006 slow-slip event, values of α do not significantly change in the sweet spot. However, there is a radical change of the LFE distribution to a strongly clustered event occurrence in the transition zone when this fault segment starts slipping. These results suggest that LFE occurrence becomes clustered when the fault interface undergoes slow slip.

**Fig. 3:**
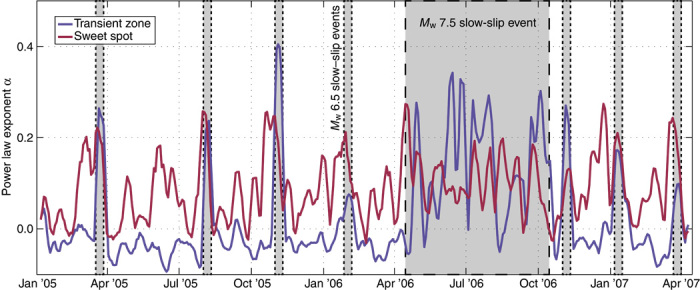
Tracking slow slip in time with the power law exponent α. The average running 10-day estimate of α is plotted for the all LFE sources in the transient zone (purple) and the sweet spot (red). Values of α increase for each of the geodetically detected slow-slip events (*6*, *18*), represented by the gray patches in the background. The dashed border for each gray patch corresponds to the slow-slip regions in Fig. 1B.

We take this hypothesis one step further and measure α in the above-described fashion in a 10-day sliding window across the full 2.5-year data set, dividing the LFE sources into the transient zone or the sweet spot based on their distance from the trench. Figure 3 shows that for every previously detected slow-slip event, including the recently reported smaller events (*18*), there is an associated increase of α in both LFE source regions. We observe that values of α in the sweet spot increase just before those in the transient zone at the beginning of every slip event, implying that each slow-slip event in Guerrero starts downdip and migrates updip. Within the sweet spot, we also note that the power law exponent α is higher than background values in between the geodetically observed slow-slip events shown in Fig. 3. This implies that there are even smaller slow-slip events within the sweet spot downdip of the recently reported 90-day slow-slip cycle (*18*) that have yet to be observed.

## DISCUSSION

Considering slow slip to be a temporally and spatially heterogeneous increase of loading rate, we can imagine a slip pulse that migrates across the LFE source region, increasing event rate within the LFE source region. If the distribution of LFEs is Poisson in the absence of slow slip, as observed in the transient zone during the inter–slow-slip period, an increased loading rate will increase the event rate but will not generate events in a clustered fashion, that is, a faster Poisson process is still Poisson.

We now consider the possibility that each LFE source analyzed here is in fact made up of multiple brittle asperities. We are only able to observe the collection of events that originate from these many different asperities within one source “patch” because of observational constraints such as frequency content and source-receiver distance. The resulting catalog of events from such a source configuration during slow slip will be made up of multiple Poisson distributions, whose combined distribution will still be Poisson because the timing of any given event will not depend on any of the other cataloged events. We therefore propose that an increased loading rate due to slow slip, even if heterogeneous in space and time, is not sufficient to explain the observed deviation from a Poisson process to a clustered one.

### Clustering through interaction

**Fig. 4:**
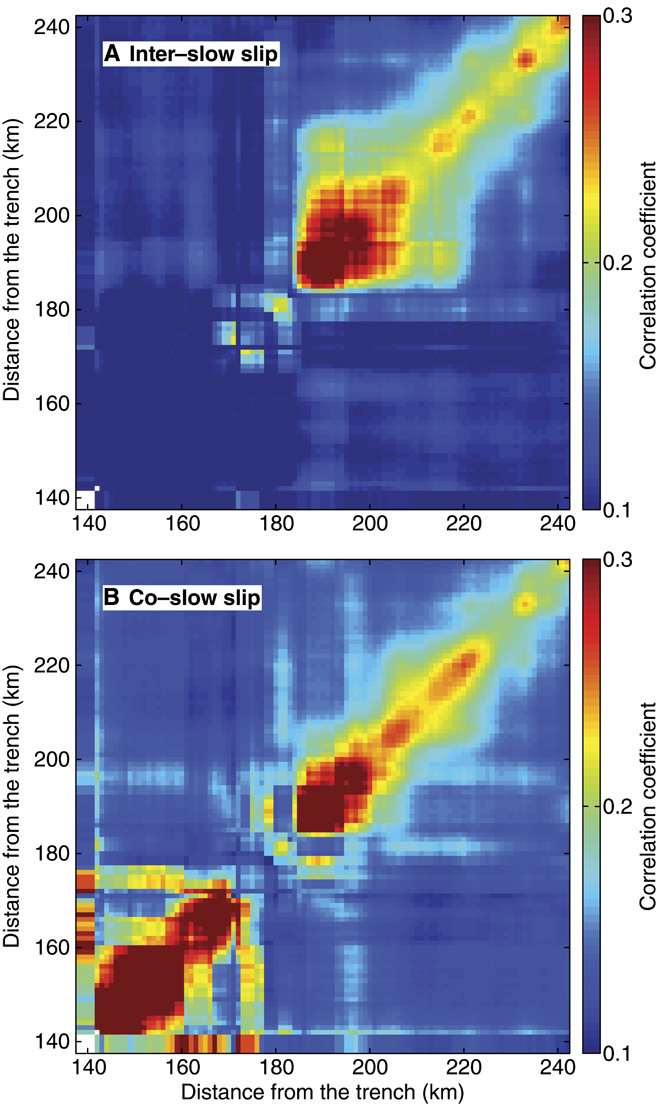
Interaction across LFE sources. (**A** and **B**) Each cell in (A) and (B) represents the spatially smoothed correlation between different LFE event counts (see text). The inter–slow-slip period is shown in (A), whereas the co–slow-slip is shown in (B). The sweet spot exhibits high levels of interaction during both time periods, whereas the transient zone is only correlated during the *M*_w_ 7.5 2006 slow-slip event (*7*).

To generate the observed clustered event distributions, we therefore propose that the asperities that emit LFEs must interact with each other, similar to how clusters of classical earthquakes form (*20*, *21*). We do not, however, implicitly implicate any physical mechanism, and only mean to refer to the correlated timing of events that originate at neighboring sources. We test this hypothesis by measuring the correlation coefficient between different LFE source event count time series, which we interpret as their level of interaction (*11*). Similar to observations of α during the two 4-month windows (Fig. 1C), we find that intersource interaction occurs all the time in the sweet spot and only during slow slip in the transient zone, as shown in Fig. 4.

The relationship between interasperity interaction and time clustering of events is confirmed by a simple conceptual model (*11*). We start with an ensemble of random Poisson processes that represent the occurrence of LFEs on independent asperities. We then introduce an interaction mechanism that accelerates future events at asperities close to a recent event. The strength of this interaction decays with the interasperity distance, and its efficiency is controlled by the effective asperity density (the number of asperities per the effective interaction distance). Figure S3 shows that when this effective asperity density increases, the simulated events start to be clustered in time. We also verify, as previously discussed, that a spatially and temporally coherent acceleration of Poisson event rates analogous to a slow-slip event cannot create the observed clustered distributions, as shown in figs. S2 and S3. Another possibility is that the slip evolution of slow slip is itself governed by a power law. We consider this, however, to be a reductive interpretation that does not provide insight on the physical processes responsible for the observed time clustering. Although we cannot prove that our model is unique, it spontaneously reproduces the deviation from a purely Poisson occurrence of events without any explicitly defined mechanism. We therefore suggest that the transition from a Poisson distribution of LFEs to the observed time clustering of events during the 2006 slow-slip event implies an increased effective asperity density.

### Possible physical mechanisms

If the interaction mechanism does not change and is constant in time, such as elastic interactions through dynamic and static stress changes, then our results imply a real increase in the number of seismically active asperities. This interpretation is, at first glance, at odds with what was found in a recent laboratory study (*22*) of a “classical” stick-slip regime where the average contact area along the fault was greatly reduced just before the rapid acceleration of slip. Therefore, the time evolution of the LFE asperity density could not be dependent on the average contact area. Instead, LFEs are the signature of the strongest contacts along the plate interface that are activated when the area around them starts creeping. The onset of slow slip would therefore reveal a dense population of strong asperities that were previously locked and that start to interact to produce the observed clustered seismicity. The other possible explanation is that a new mechanism, which is latent while the plate interface is locked, dominates the interaction between asperities during slow slip. Growing reports of high pore-fluid pressure in the source regions of slow-slip events, tectonic tremor, and LFEs (*2*, *23*, *24*), along with recent seismological evidence of migrating pore pressure pulses during slow slip (*8*) and geological observations of along-fault hydrofracturing colocated with slow slip in an exhumed subduction zone (*25*), suggest that dynamic changes in pore pressure during slip could play a role.

## CONCLUSIONS

Regardless of the mechanism, our observations lead to a conceptually new interpretation of the relationship between slow slip and LFEs, and impose new constraints on future mechanical and numerical models of the slow-slip cycle. We show that bursts of LFEs are more than just the amalgam of events on independent asperities activated by simple pulses of slow slip. LFE asperities are in fact active all the time, and their behavior depends on the state of the fault contact along the interface. When the fault is locked or creeping at slip rates much slower than slow slip, every asperity acts independently of the others and the resulting event occurrence is Poisson. However, when the interface starts slipping, interaction between individual asperities appears to be facilitated and results in a collective behavior that produces a time-clustered event distribution. Using the point process statistical analysis described, we are able to directly measure whether this clustering occurs.

Finally, we note that the observed behavior of LFE asperities during the slow-slip cycle is similar to the known space-time evolution of regular earthquakes. The seismic cycle of typical earthquakes consists of fast slip followed by a long quiescence during stress accumulation. This quiescence is not absolute, and a number of smaller events typically occur in a Poisson fashion. In contrast, rapid slip during the rupture of the large earthquakes that define the seismic cycle shows a clear clustering in high-frequency radiation. Such clustering has been suggested to represent organized pulses from interacting asperities (*26*). This parallel between “fast” and “slow” earthquakes implies a universal clustered rupture process that operates over very different scales in both time and space.

## MATERIALS AND METHODS

### Cataloging LFEs in Guerrero

The dense catalog of more than 1.8 million LFEs spread over the 1120 different sources that we analyzed here was generated through a systematic search for repeating events along the Guerrero subduction interface (*5*). The detection method consists of two steps, the first of which is a source scanning algorithm (*10*) that back-projects the seismic energy computed from continuous seismic records on a grid of potential source locations and detects the “bright spots” in the resulting time-space energy distributions. Each of the detected events is then used as a template in a network-based matched-filter search. During this stage, the template waveforms are correlated in a sliding window across the continuous records of different stations and components to look for events whose waveforms are significantly similar to those of the template. With a large enough seismic network, such a detection method can identify events hidden within the ambient noise. The family of events detected using a given template (seismic multiplets) represents the seismicity originating from a single source location. The stacked waveforms from all multiplets of a single family have an increased signal-to-noise ratio and are used to locate the source position.

### Verifying inter–slow-slip LFEs are not false detections

To verify that the inter–slow-slip events are not false detections, we compared their stacked waveforms with those of the co–slow-slip events, as shown in fig. S1. As described in the work that produced the event catalog analyzed here (*5*), only a subset of stations was used in the matched-filter search for every particular LFE source (family of multiplets). We then stacked the waveforms from the time windows containing the detections at all stations, including those that were not used in the matched-filter search. With this approach, the false detections, such as random matches of the template, could result in a constructive stack at stations used in the matched-filter search. However, such random matching should not contain coherent arrivals at stations not used in the template comparison. The emergence of coherent arrivals at these stations clearly indicate that the ensemble of the inter–slow-slip events is not dominated by false detections and corresponds to the seismic energy emitted by the same LFE sources that are acting during the slow slip.

### Intersource correlation of event count time series

Earthquake clustering, both in time (*21*) and in space (*20*), is typically associated with interactions between events through dynamic and static stress perturbations, such as the aftershocks that are triggered following a mainshock. Similarly, the observed LFE clustering can be caused by the interaction of LFEs occurring on different asperities. To quantify the level of possible interasperity interaction, we evaluated the level of correlation between different LFE sources by computing correlation coefficients between pairs of event count signals (*27*, *28*). Results of this analysis for the inter– and co–slow-slip periods after applying a two-dimensional (2D) 10-km spatial smoothing are shown in Fig. 4. The space-time variations of the intersource correlations are very similar to those of the power law exponent α in Figs. 1C and 3, with strong clustering in the sweet spot and transient zone during slow slip.

### Conceptual model of the time clustering of events caused by interasperity interaction

We uniformly distributed *I* point processes along a 1D interface of length *X* with the *i*th process located at *x*_*i*_. Each point process represents the seismic cycle of an asperity, generating events whose recurrence times are drawn as waiting times from a Poisson distribution. After running a simulation for *t*_max_ time and generating *E*_total_ events, the model’s output that we analyzed is the catalog of event times *t*_e_ at some asperity *i*. The model time *t* is continuous and discretely sampled with a flexible time step detailed below.

At the start of the model simulation, we draw the waiting time *w*_*i*_ for the next event for each asperity *i* as

$$w_{i}=\frac{-\text{ln}\;R}{\lambda }$$ (1)

where *R* is a random number drawn from a uniform distribution between 0 and 1, and λ is the average event rate. Initializing the next event time τ_*i*_ at time *t*_*i*_, we have

$$\tau_{i}=w_{i}+t_{i}$$ (2)

Starting from any time *t*, we can then advance to the next event at asperity *i* with a time step of Δ*t*_*i*_

$$\Delta t_{i}=\tau_{i}-t$$ (3)

When *t* = τ_*i*_, a new event *e* is generated at asperity *i* and logged at time *t*_*e*_ = *t* and position *x*_*e*_ = *x*_*i*_ with waiting time *w*_*e*_ = *w*_*i*_. The next event time τ_*i*_ is then computed at *t*_*i*_ = *t*_*e*_ after drawing a new *w*_*i*_ from Eq. 1.

A deviation from a purely Poisson behavior is brought into the model by introducing a clock advance at each asperity, *C*_*i*_(*t*), that reduces the amount of time until the next event

$$\Delta t_{i}=\frac{\tau_{i}-t}{C_{i}(t)+1}$$ (4)

We define *C*_*i*_(*t*) to always be greater than or equal to zero. As long as *C*_*i*_(*t*) is constant [that is, *C*_*i*_(*t*) = *C*_*i*_], we can efficiently advance the model time to the next event across the entire interface using the smallest Δ*t*_*i*_ as the time step.

The first term of the clock advance *C*_*i*_(*t*) is *A*_*i*_ (*t*,*e*), the acceleration of the neighboring point processes following an event that simulates asperity interaction

$$A_{i}(t,e)=\{\begin{array}{cc} x_{c}{\left(\lambda {\left|x_{i}-x_{e}\right|}^{2}\right)}^{-1}, & \text{if}\;x_{i}\neq x_{e}\;\text{and}\;t_{e}\leq t\leq t_{e}+\gamma \\ 0, & \text{otherwise} \end{array}$$ (5)

where *E* is the number of events that have occurred so far, *x*_c_ is the critical interaction distance, and γ is the interaction time scale. We used a critical distance *x*_c_ of 1 to keep the model as simple as possible. We have designed *A*_*i*_(*t*,*e*) to induce a clock advance equal to the average waiting time λ^−1^ of the interface in a constant fashion over a time window of length γ.

In addition to the interaction term *A*_*i*_(*t*,*e*), we also consider a second term *P*_*i*_(*t*) that represents a spatially and temporally heterogeneous clock advance, analogous to a slow slip pulse that migrates across the model space and locally increases the event rate. We introduce this into the model as *P*_*i*_(*t*), the clock advance due to a migrating boxcar pulse of width 2*W* and traveling from position $x_{p}^{0}$ at velocity *V*

$$P_{i}(t)=\{\begin{array}{cc} p, & \text{if}\;x_{p}(t)-W\leq x_{i}\leq x_{p}(t)+W\\ 0, & \text{otherwise} \end{array}$$ (6)

$$x_{p}(t)=x_{p}^{0}+Vt$$ (7)

where *p* is the “height” of the boxcar and is the clock advance induced at all asperities within the pulse, and *x*_*p*_(*t*) is the position of the center of the pulse at time *t*.

The clock advance *C*_*i*_(*t*) is therefore defined as

$$C_{i}(t)=\sum_{e=1}^{E}A_{i}(t,e)+P_{i}(t)$$ (8)

The clock advance is a step function that is not smoothly varying. It is, however, dependent on time, and Eq. 4 is only valid while *C*_*i*_(*t*) does not change. It is then trivial to calculate the amount of time until *C*_*i*_(*t*) changes due to a change in the interaction term *A*_*i*_(*t*,*e*)

$$\Delta t^{E}=e=1E\text{min}\;\Delta t^{e}$$ (9)

$$\Delta t^{e}=\{\begin{array}{cc} t_{e}+\gamma -t, & \text{if}\;t_{e}+\gamma >t\\ t_{\text{max}}, & \text{otherwise} \end{array}$$ (10)

or the migrating pulse term *P*_*i*_(*t*)

$$\Delta t^{P}=i=1I\text{min}\Delta t_{i}^{p}$$ (11)

$$\Delta t_{i}^{p}=\{\begin{array}{cc} {}[x_{i}-(x_{p}(t)+W)]V^{-1}, & \text{if}\;x_{i}>x_{p}(t)+W\\ {}[x_{i}-(x_{p}(t)-W)]V^{-1}, & \text{if}\;x_{p}(t)-W\leq x_{i}\leq x_{p}(t)+W\\ t_{\text{max}}, & \text{otherwise} \end{array}$$ (12)

We can therefore determine the maximum time step possible while *C*_*i*_ (*t*) stays constant by using the smallest time step among Δ*t*_*i*_, Δ*t*^*E*^, and Δ*t*^*P*^. In such a way, we can efficiently advance through time until *t*_max_. Because of this flexible time stepping method, the event timing of the resulting catalog is not on a discretized time scale, and the full spectrum of recurrence times is possible.

After an initial exploration of the basic parameter space without a slow slip–like pulse (*p* = 0), we find that the parameter that changes the observed behavior of the resulting synthetic event catalog is the effective asperity density (the number of asperities per the critical interaction distance *x*_c_). Fixing a number of parameters (the full set is shown in table S1), we first simulate a model with 20 asperities whose events are distributed in a random “cloud” of points on a recurrence interval plot, as shown in fig. S2 (A and B). If, however, we sufficiently increase the effective asperity density, events will start to accelerate neighboring asperities, increasing the likelihood that events occur both faster than the background Poisson event rate λ and in a correlated fashion. This produces the clustering that is seen in fig. S2 (A and B) that qualitatively looks very similar to what is seen in the real data set in Fig. 2. We compared the results of our conceptual model to other recent numerical models (*29*–*31*) to look for similar deviations from a Poisson process during slip processes, but we did not find any obvious similarities.

We then investigated the effect of the slow slip–like pulse term, *P*_i_(*t*) by dividing each model run into two time periods: a co–slow-slip period while the migrating pulse still lies along the model space (*t* < 10,000) and an inter–slow-slip period after it has left the model space (*t* > 10,000). We were then able to measure the power law exponent α for each time period and compare them while testing different effective asperity densities, as shown in fig. S3. We found that whereas there are more events during the co–slow-slip period, they are generated in a Poisson fashion and their timing is not correlated with neighboring asperities. This is not unexpected because both a faster Poisson process is still Poisson and coherently accelerated Poisson processes still emit events in an uncorrelated random fashion. We therefore suggest that the accelerated slip rate due to a migrating slow-slip pulse is not sufficient to generate the observed clustered LFE distributions.

### Stability of point process analysis

We reproduced the autocorrelation and spectrum shown in Fig. 2 (C and D) using different window lengths and bin widths in figs. S4 to S7 to verify the stability of point process analysis. We found that as long as a reasonable window length and bin width are chosen, the estimated power law exponent is stable. This is not surprising given the fact that, by definition, a power law exponent does not have a characteristic time scale, and our analysis should therefore be insensitive to the time scales used to estimate the event count time series.

## Supplementary Material

http://advances.sciencemag.org/cgi/content/full/2/4/e1501616/DC1

## SUPPLEMENTARY MATERIALS

Supplementary material for this article is available at http://advances.sciencemag.org/cgi/content/full/2/4/e1501616/DC1 fig. S1. Stacked waveforms of a transient zone LFE source during the inter– (black) and co–slow-slip (red) time periods. fig. S2. Three synthetic catalogs from our numerical model. fig. S3. Parametric estimation of the power law exponent α with and without slow slip. fig. S4. Stability of event count time series autocorrelation with respect to analyzed window duration. fig. S5. Stability of event count time series spectrum with respect to analyzed window duration. fig. S6. Stability of event count time series autocorrelation with respect to analyzed bin width. fig. S7. Stability of event count time series spectrum with respect to analyzed bin width. table S1. Numerical model parameters used in figs. S2 and S3.
