# Supplementary material for: The evolving interaction of low-frequency earthquakes during transient slip
Source: Sci Adv. 2016 Apr 22;2(4):e1501616. doi: 10.1126/sciadv.1501616 (PMC4846440; doi:10.1126/sciadv.1501616)
Supplement: http://advances.sciencemag.org/cgi/content/full/2/4/e1501616/DC1 [file supp_2_4_e1501616__index.html]

Science Advances | Science Advances

## Supplementary Materials

**This PDF file includes:**

- fig. S1. Stacked waveforms of a transient zone LFE source during the inter–(black) and co–slow-slip (red) time periods.
- fig. S2. Three synthetic catalogs from our numerical model.
- fig. S3. Parametric estimation of the power law exponent α with and without slow slip.
- fig. S4. Stability of event count time series autocorrelation with respect to analyzed window duration.
- fig. S5. Stability of event count time series spectrum with respect to analyzed window duration.  
   fig. S6. Stability of event count time series autocorrelation with respect to analyzed bin width.
- fig. S7. Stability of event count time series spectrum with respect to analyzed bin width.
- table S1. Numerical model parameters used in figs. S2 and S3.

Download PDF

**Files in this Data Supplement:**

- Adobe PDF - 1501616\_SM.pdf
