# Supplementary material for: The evolving interaction of low-frequency earthquakes during transient slip
Source: Sci Adv. 2016 Apr 22;2(4):e1501616. doi: 10.1126/sciadv.1501616 (PMC4846440; doi:10.1126/sciadv.1501616)
Supplement: http://advances.sciencemag.org/cgi/content/full/2/4/e1501616/DC1 [file 1501616_SM.pdf]

Science Advances

AAAS

advances.sciencemag.org/cgi/content/full/2/4/e1501616/DC1

Supplementary Materials for

The evolving interaction of low-frequency earthquakes during transient slip

William B. Frank, Nikolaï M. Shapiro, Allen L. Husker, Vladimir Kostoglodov, Alexander A. Gusev, Michel Campillo

Published 22 April 2016, *Sci. Adv.* **2**, e1501616 (2016)
DOI: 10.1126/sciadv.1501616

The PDF file includes:

- fig. S1. Stacked waveforms of a transient zone LFE source during the inter– (black) and co–slow-slip (red) time periods.
- fig. S2. Three synthetic catalogs from our numerical model.
- fig. S3. Parametric estimation of the power law exponent α with and without slow slip.
- fig. S4. Stability of event count time series autocorrelation with respect to analyzed window duration.
- fig. S5. Stability of event count time series spectrum with respect to analyzed window duration.
- fig. S6. Stability of event count time series autocorrelation with respect to analyzed bin width.
- fig. S7. Stability of event count time series spectrum with respect to analyzed bin width.
- table S1. Numerical model parameters used in figs. S2 and S3.

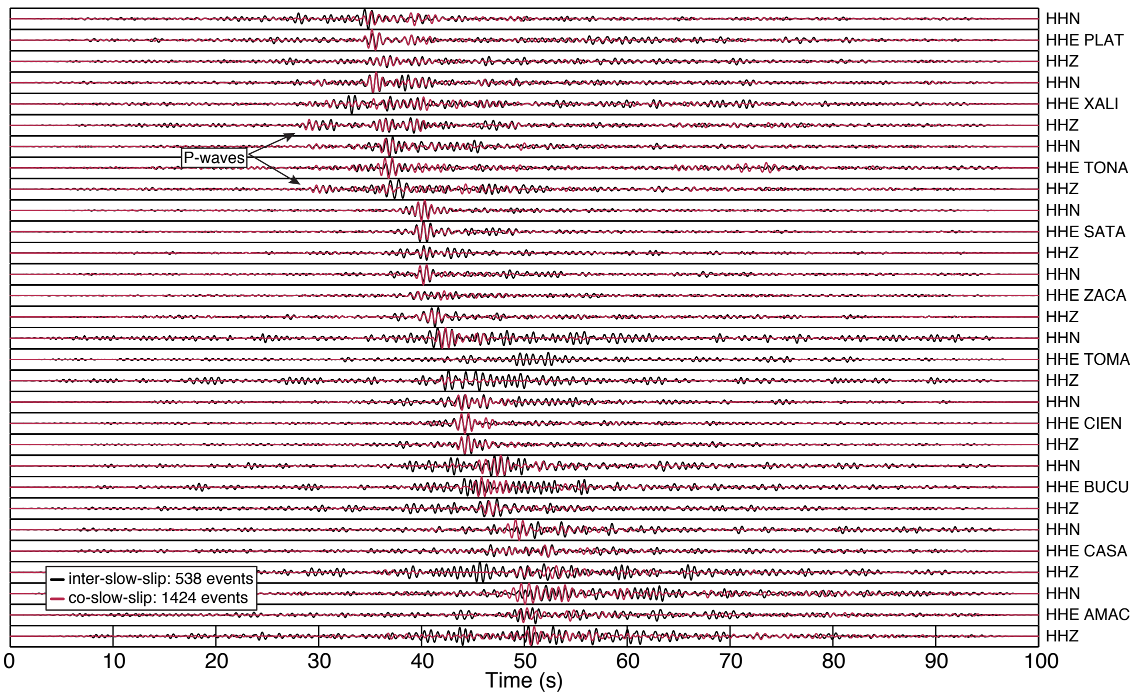

fig. S1. Stacked waveforms of a *transient zone* low-frequency earthquake (LFE) source during the inter- (black) and co-slow slip (red) time periods. All stations but AMAC were used to detect this LFE source. Both event catalogs reproduce the same arrivals and phases, confirming that though randomly distributed (see Fig. 2), the inter-slow slip events are not false detections.

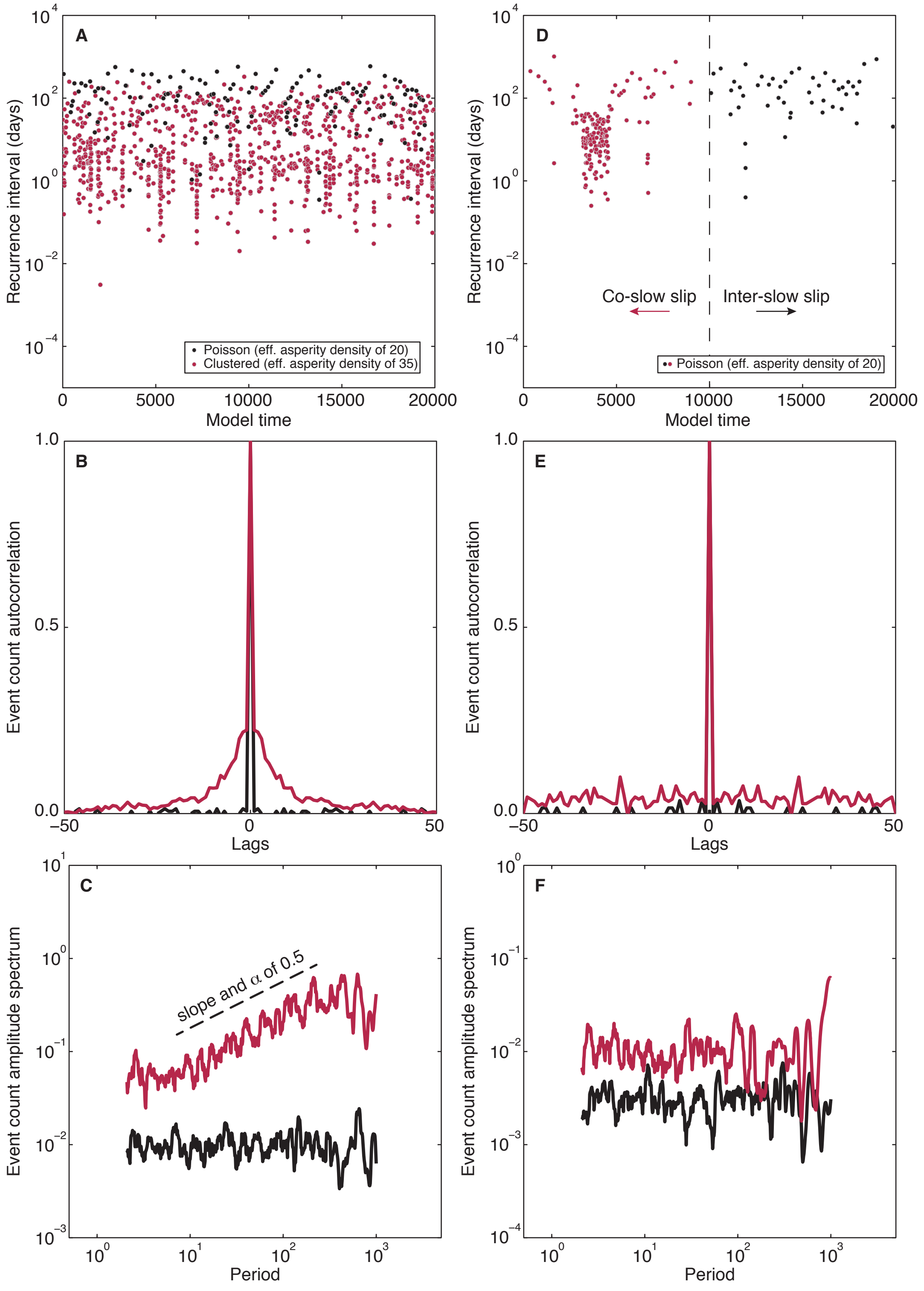
A
Recurrence interval (days)
Model time
Poisson (eff. asperity density of 20)
Clustered (eff. asperity density of 35)
D
Co-slow slip
Inter-slow slip
Poisson (eff. asperity density of 20)
B
Event count autocorrelation
Lags
E
C
Event count amplitude spectrum
slope and α of 0.5
Period
F

fig. S2

fig. S1. Three synthetic catalogs from our numerical model. **A** and **D** show the recurrence intervals for model simulations over 20000 time units, defined as the elapsed time between successive events in a given catalog. The event count autocorrelation (see text) of each respective catalog is shown in **B** and **E** while their spectra are shown in **C** and **F**. Panels A, B, and C show two synthetic catalogs who have different effective asperity densities: black represents the synthetic catalog (20 asperities) whose asperities do not significantly interact while red represents the synthetic catalog (35 asperities) that exhibits interaction and a clear collective behavior. Panels D, E, and F show a synthetic catalog with an effective asperity density of 20 that undergoes a migrating clock advance pulse analogous to a slow slip event. Separately analyzing the co- (red) and inter-slow slip (black) time periods for the same asperity, we do not observe any change in the level of clustering due to the migrating pulse.

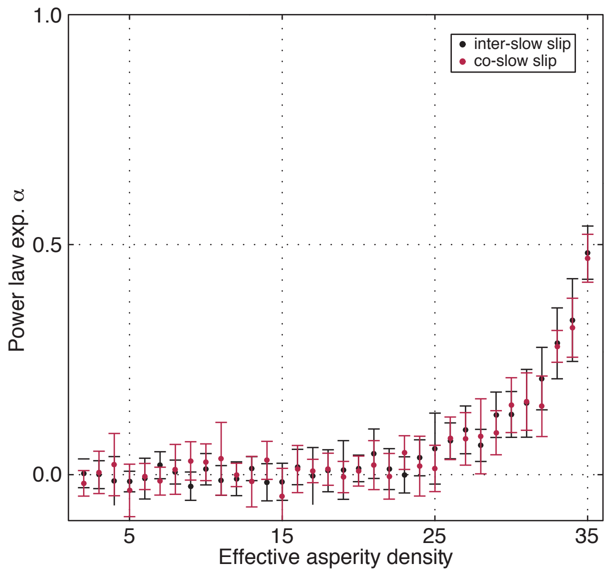

fig. S2. Parametric estimation of the power law exponent α with and without slow slip. Each point represents the median value of eight estimations of α for a model simulation with a given number of asperities; an error of 1σ is plotted for each point. Each model simulation is divided into two time periods (see Fig. S1): points in black represent the power law exponent α during the inter-slow slip period after the pulse as has left the model space and points in red represent the co-slow slip period while the pulse travels across the model space.

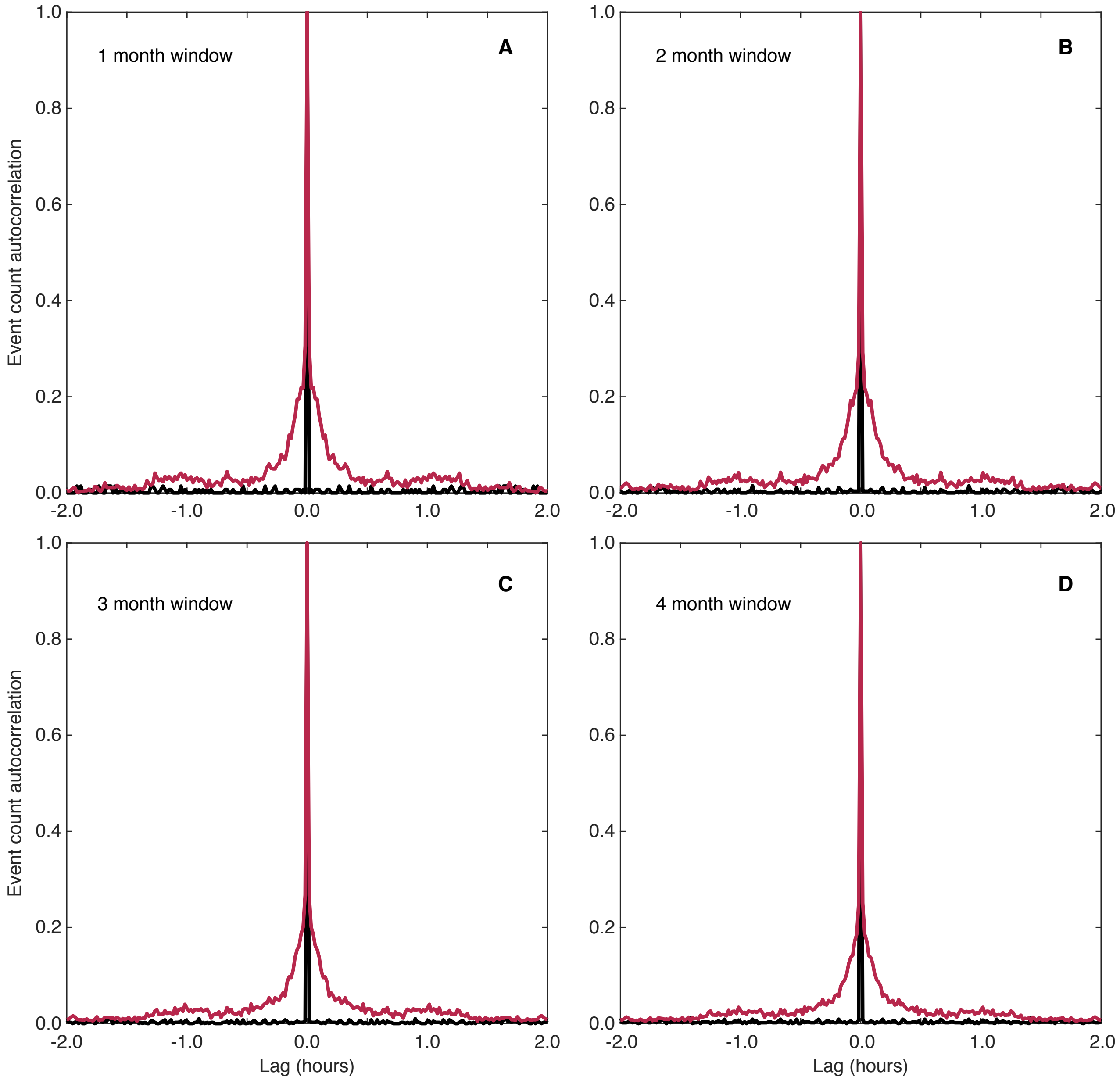

fig. S3. Stability of event count time series autocorrelation with respect to analyzed window duration. Each panel represents the event count time series autocorrelation (see text) for different window durations: 1 month (**A**), 2 months (**B**), 3 months (**C**), and 4 months (**D**; used in study).

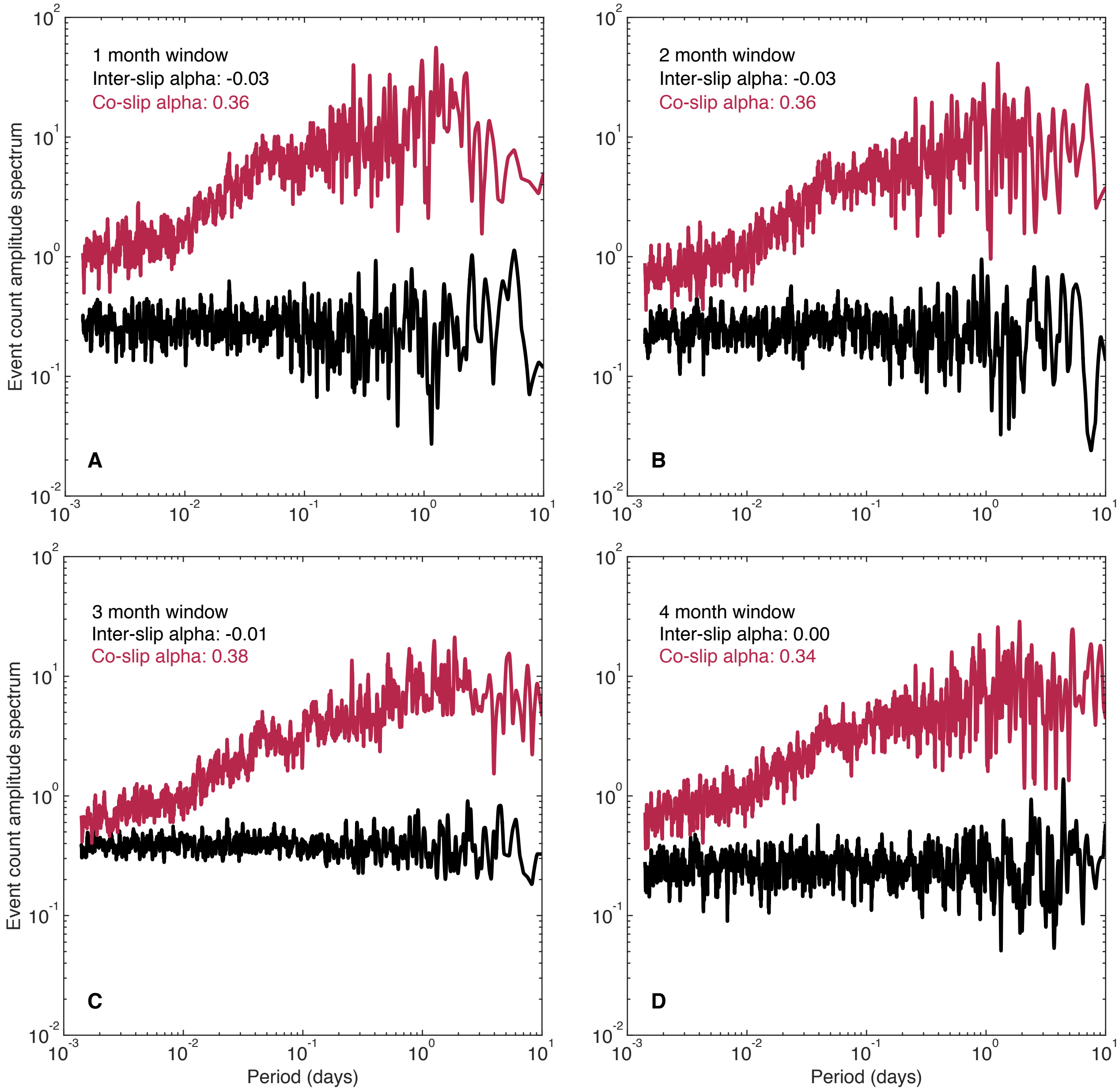

fig. S1. Stability of event count time series spectrum with respect to analyzed window duration. Each panel represents the spectrum of the event count time series autocorrelation (see text) for different window durations: 1 month (**A**), 2 months (**B**), 3 months (**C**), and 4 months (**D**; used in study).

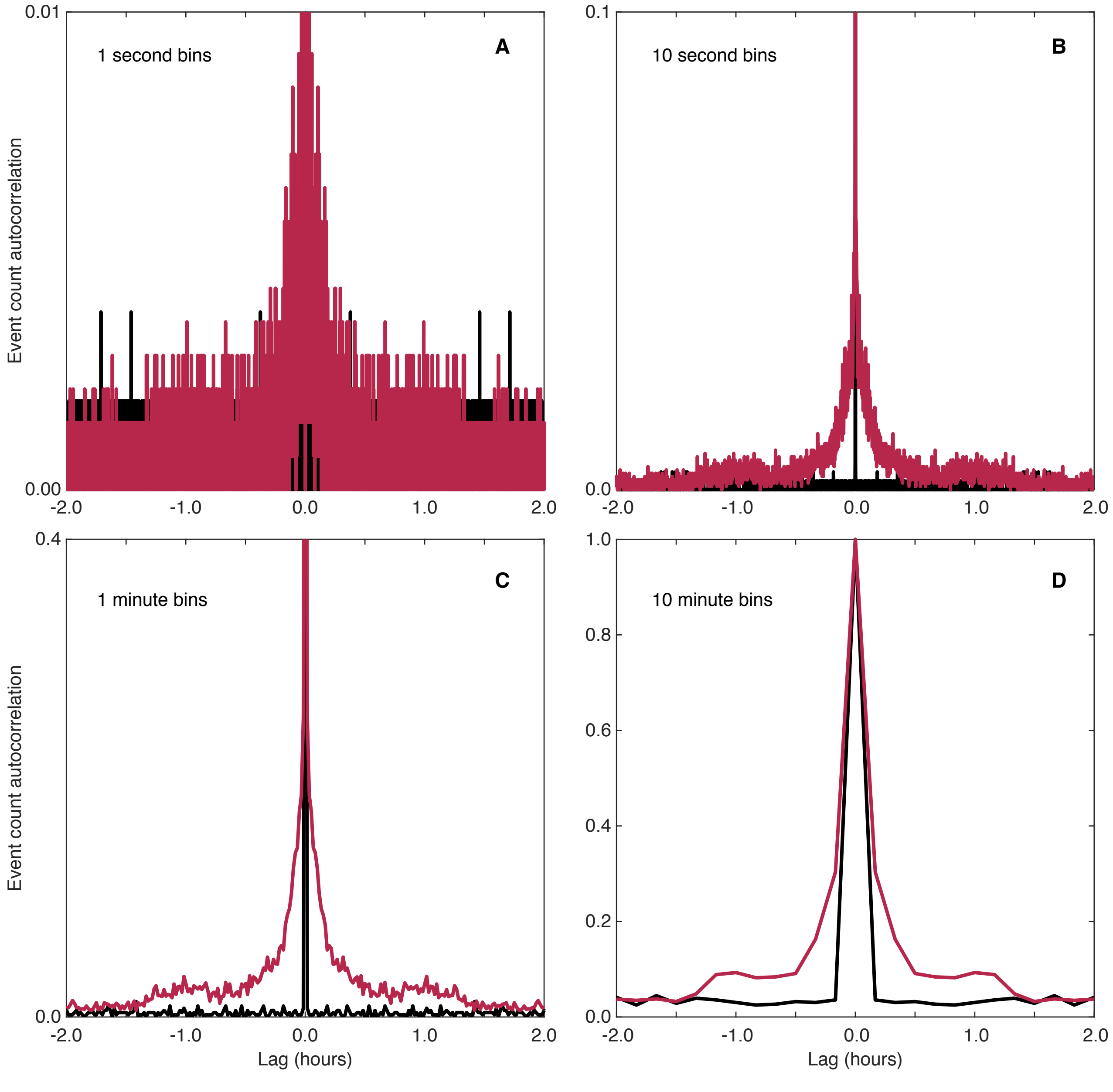

fig. S5. Stability of event count time series autocorrelation with respect to analyzed bin width. Each panel represents the event count time series autocorrelation (see text) for different bin widths: 1 second (**A**), 10 seconds (**B**), 1 minute (**C**; used in study), and 10 minutes (**D**).

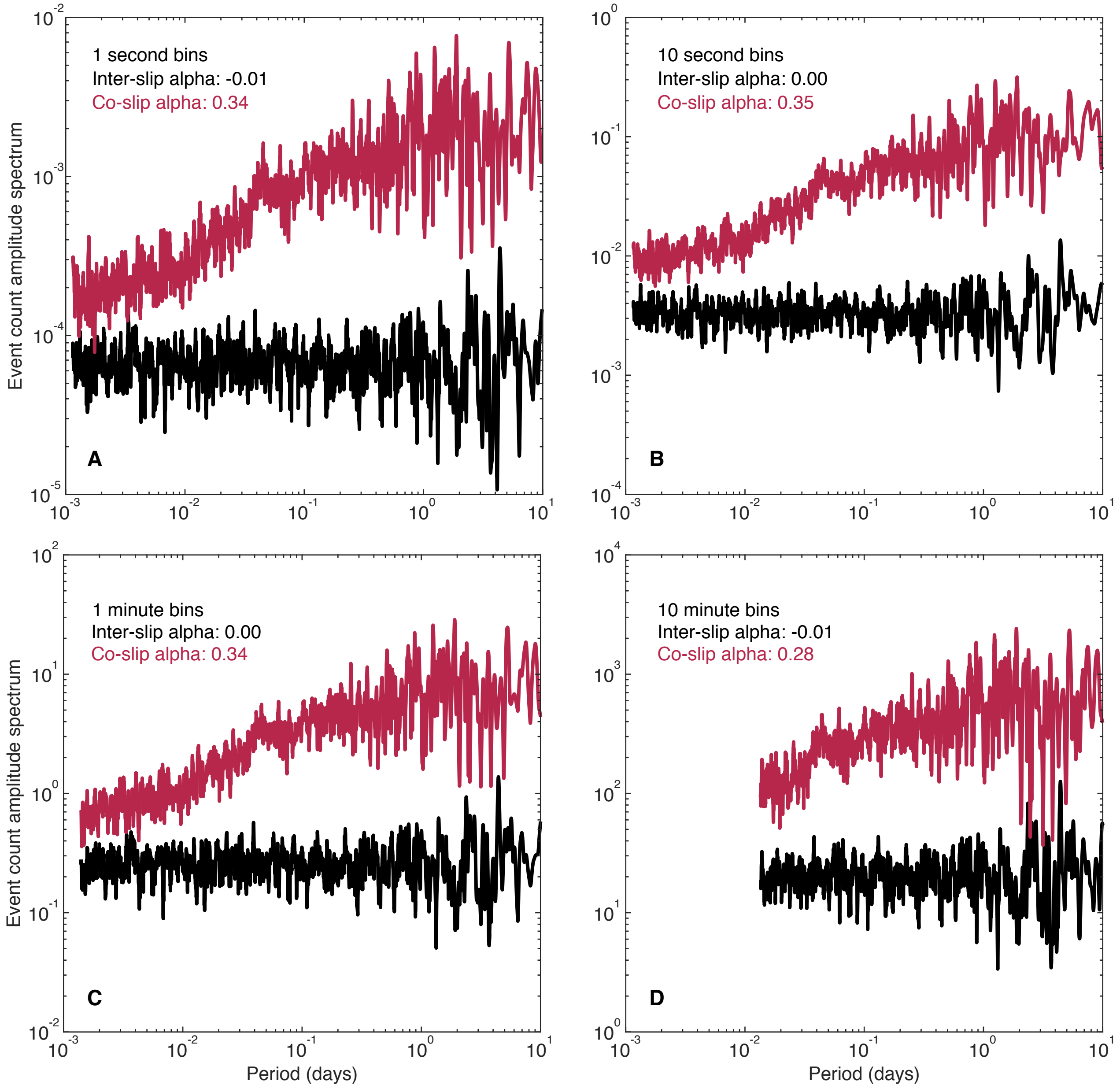

fig. S6. Stability of event count time series spectrum with respect to analyzed bin width. Each panel represents the spectrum of the event count time series autocorrelation (see text) for different bin widths: 1 second (**A**), 10 seconds (**B**), 1 minute (**C**; used in study), and 10 minutes (**D**.

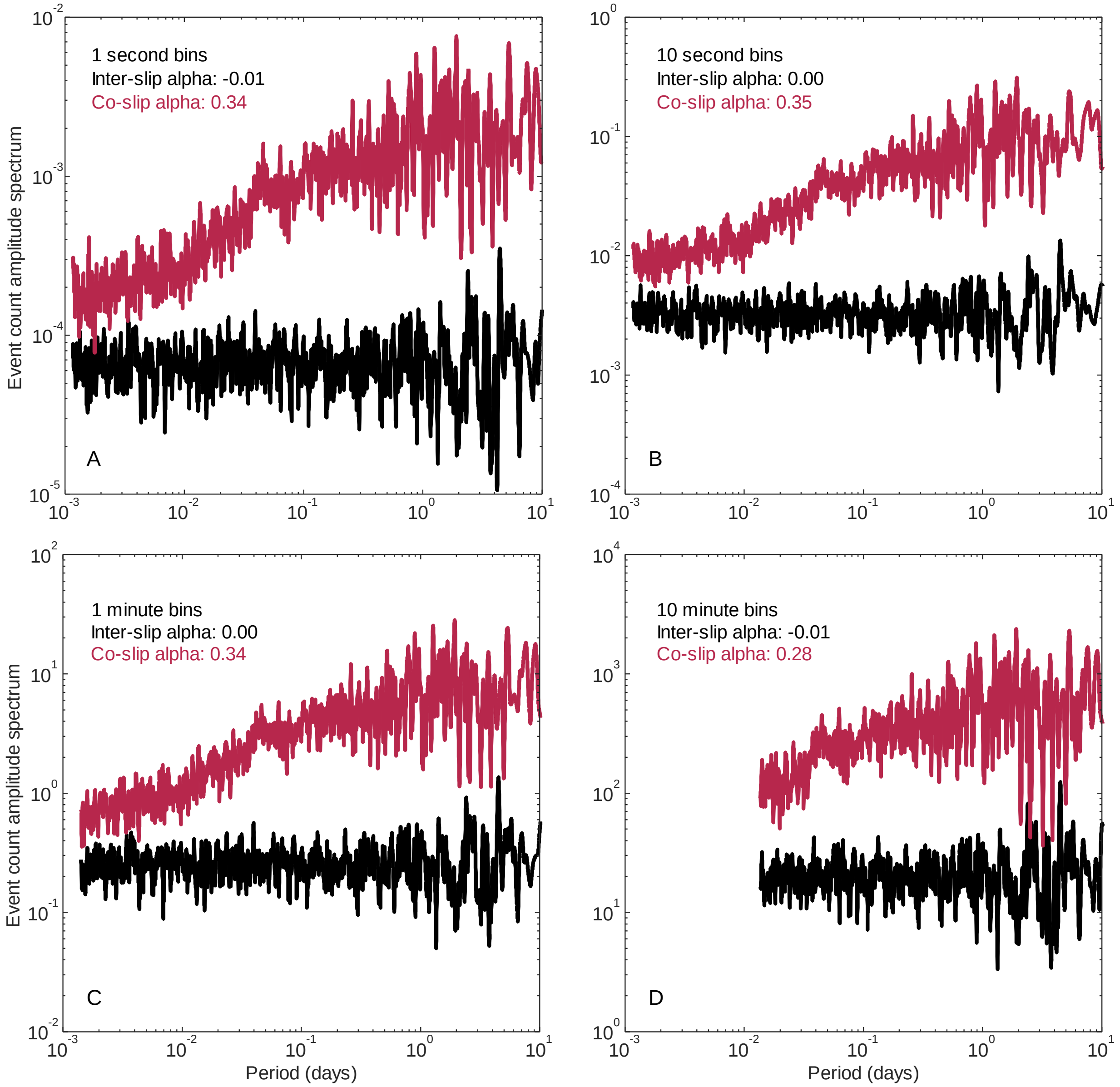

fig. S7. Stability of event count time series spectrum with respect to analyzed bin width.

table S1.Numerical model parameters used in figs. S1 and S2.

Parameter	Symbol	Value
Number of asperities	I	$[2, 35]$
Interface length	X	100
Max time	t_{max}	20000
Random uniform variable	R	$[0, 1]$
Average Poisson event rate	λ	$50 \cdot 10^{-3}$
Critical interaction distance	x_c	1
Interaction time scale	γ	2.5
Initial pulse position	x_p^0	-50
Pulse width	$2W$	30
Pulse height (clock advance)	p	10
Pulse migration velocity	V	0.02
